# Characterization of vaginal microbiomes in clinician-collected bacterial vaginosis diagnosed samples

**DOI:** 10.1128/spectrum.02582-24

**Published:** 2025-02-25

**Authors:** Hayden N. Brochu, Qimin Zhang, Kuncheng Song, Ling Wang, Emily A. Deare, Jonathan D. Williams, Crystal R. Icenhour, Lakshmanan K. Iyer

**Affiliations:** 1 Labcorp Center for Excellence in Data Science, AI and Bioinformatics, Burlington, North Carolina, USA; 2 Labcorp Research and Development, Office of the CSO, Burlington, North Carolina, USA; Chengdu University, Chengdu, Sichuan, China

**Keywords:** bacterial vaginosis, vaginal microbiome, 16S rRNA

## Abstract

Bacterial vaginosis (BV) is a type of vaginal inflammation caused by bacterial overgrowth, upsetting the healthy microbiome of the vagina. Existing clinical testing for BV is primarily based upon physical and microscopic examination of vaginal secretions. Modern PCR-based clinical tests target panels of BV-associated microbes, such as the Labcorp NuSwab test that targets *Atopobium* (*Fannyhessea*) *vaginae*, *Megasphaera-1*, and *Bacterial Vaginosis Associated Bacterium (BVAB)−2*. Remnant clinician-collected NuSwab vaginal swabs underwent DNA extraction and 16S V3–V4 rRNA gene sequencing to profile microbes in addition to those included in the Labcorp NuSwab test. Community state types (CSTs) were determined using the most abundant taxon detected in each sample. PCR results for NuSwab panel microbial targets were compared against the corresponding microbiome profiles. Metabolic pathway abundances were characterized via metagenomic prediction from amplicon sequence variants (ASVs). 16S V3–V4 rRNA gene sequencing of 75 remnant vaginal swabs yielded 492 unique 16S V3–V4 ASVs, identifying 83 unique genera. NuSwab microbe quantification was strongly concordant with quantification by sequencing (*P* < 0.01). Samples in CST-I (18 of 18, 100%), CST-II (three of three, 100%), CST-III (15 of 17, 88%), and CST-V (one of one, 100%) were largely categorized as BV-negative via the NuSwab panel, while most CST-IV samples (28 of 36, 78%) were BV-positive or BV-indeterminate. BV-associated microbial and predicted metabolic signatures were shared across multiple CSTs. These findings highlight robust sequencing-based quantification of Labcorp NuSwab BV microbes, accurate discrimination of vaginal microbiome CSTs dominated by distinct *Lactobacilli*, and expanded the identification of BV-associated bacterial and metabolic biomarkers.

**IMPORTANCE:**

Bacterial vaginosis (BV) poses a significant health burden for women during reproductive years and onward. Current BV diagnostics rely on either panels of select microbes or on physical and microscopic evaluations by technicians. Here, we sequenced the microbiome profiles of samples previously diagnosed by the Labcorp NuSwab test to better understand disruptions to the vaginal microbiome during BV. We show that microbial sequencing can faithfully reproduce targeted PCR diagnostic results and can improve our knowledge of healthy and BV-associated microbial and metabolic biomarkers. This work highlights a robust, agnostic BV classification scheme with potential for future development of sequencing-based BV diagnostic tools.

## INTRODUCTION

Women’s health is an issue of global concern that requires substantial effort to resolve the gender inequities in the funding and quality of care that persist today (1, 2). The World Health Organization (WHO) has recognized the importance of women’s health and is investing considerable resources and efforts to enhance access to healthcare for women (3). Among the health challenges faced by women, bacterial vaginosis (BV) poses a significant health burden with a prevalence of 21.2 million women aged 14–49 as of 2004, occurring initially during reproductive years and continuing through life (4). Healthy vaginal microbiomes can contain dozens of microbial species in a specific balance (5, 6). Subtle variations in the vaginal microbiota, particularly among *Lactobacillus* species, have been associated with female reproductive health (7). BV is a type of vaginal inflammation caused by bacterial overgrowth that upsets the healthy microbiome of the vagina, which often leads to elevated vaginal pH and symptoms of abnormal discharge and odor (8).

Existing clinical testing for BV is primarily based upon physical and microscopic examination of vaginal secretions, such as Amsel’s criteria and Nugent score (9). In Amsel’s criteria, the presence of at least three out of the following four criteria is indicative of BV: high vaginal pH (>4.5), thin and homogenous discharge, malodorous fishy discharge upon adding 10% potassium hydroxide, and identification of clue cells. The Nugent score is a scoring system that evaluates the presence of *Lactobacillus spp*. morphotypes, *Gardnerella vaginalis* morphotypes, and *Mobiluncus spp*. morphotypes. Newer nucleic acid-based clinical tests are now available. The Labcorp NuSwab test (10, 11) is a highly sensitive and specific PCR-based method that diagnoses BV in symptomatic women by quantifying three key BV-associated microbes: *Atopobium* (*Fannyhessea*) *vaginae*, *Bacterial Vaginosis Associated Bacterium (BVAB)−2*, and *Megasphaera-1*. While these diagnostics provide widely accessible clinical assessment of BV, many symptomatic women receive negative test results (roughly 50% in some studies) and have limited healthcare alternatives for further diagnostic evaluation and treatment. Some of these women test positive on broader panel vaginitis tests (e.g., NuSwab Vaginitis Plus) that also check for other common sources of vaginal inflammation, such as *Candida spp*. and *Trichomonas vaginalis*. However, many women fail to receive a diagnosis and often face negative impacts on their quality of life and mental health (12), so it is imperative to expand and improve BV diagnostic tools available to clinicians.

16S rRNA gene sequencing, an amplicon-based next-generation sequencing approach, is a more comprehensive method for characterizing the composition and diversity of vaginal microbiomes. Such sequencing-based analyses have drastically improved our understanding of vaginal microbiome diversity and the shifts in microbiome composition throughout a woman’s premenarchal and reproductive years (13–16). Amplicon sequencing-based profiling has also revealed unique community state types (CSTs) within the vaginal microbiome, which are commonly defined by the key *Lactobacillus* species detected (14). *Lactobacilli* are essential for vaginal microbiome health, interacting with mucosal immune and epithelial cells via immunomodulatory mechanisms (17, 18). Disruptions to this *Lactobacillus*-mediated vaginal homeostasis have been associated with dysbiotic conditions, namely, BV and aerobic vaginitis (19). Many amplicon sequencing-based investigations of vaginal microbiomes have identified additional correlates of BV positivity, such as *Gardnerella* and *Prevotella* (20–22). Despite these advances in our understanding of BV-associated vaginal microbiota, testing of clinical samples using PCR-based diagnostics provides limited clinical utility (23).

In this study, we characterized the vaginal microbiomes of 75 clinician-collected remnant NuSwab vaginal swabs via 16S V3–V4 rRNA gene sequencing. We elucidated the rich diversity and CSTs of these vaginal microbiomes with amplicon sequence variant (ASV) resolution, showing that BV typically manifests in *Lactobacillus*-deficient microbiome compositions. We also showed that amplicon-based sequencing accurately identifies the three microbes targeted by the NuSwab BV PCR assay, as well as identifying additional BV-associated microbes. Using metabolic predictions from these ASVs, we identified metabolic pathways significantly associated with BV positivity across multiple CSTs. These findings show that amplicon sequencing can accurately reproduce PCR-based testing results and facilitate biomarker discovery for the enhancement of BV diagnostic testing capabilities.

## RESULTS

### 16S rRNA gene sequencing accurately reproduces PCR-based BV diagnostic testing results

**Fig 1:**
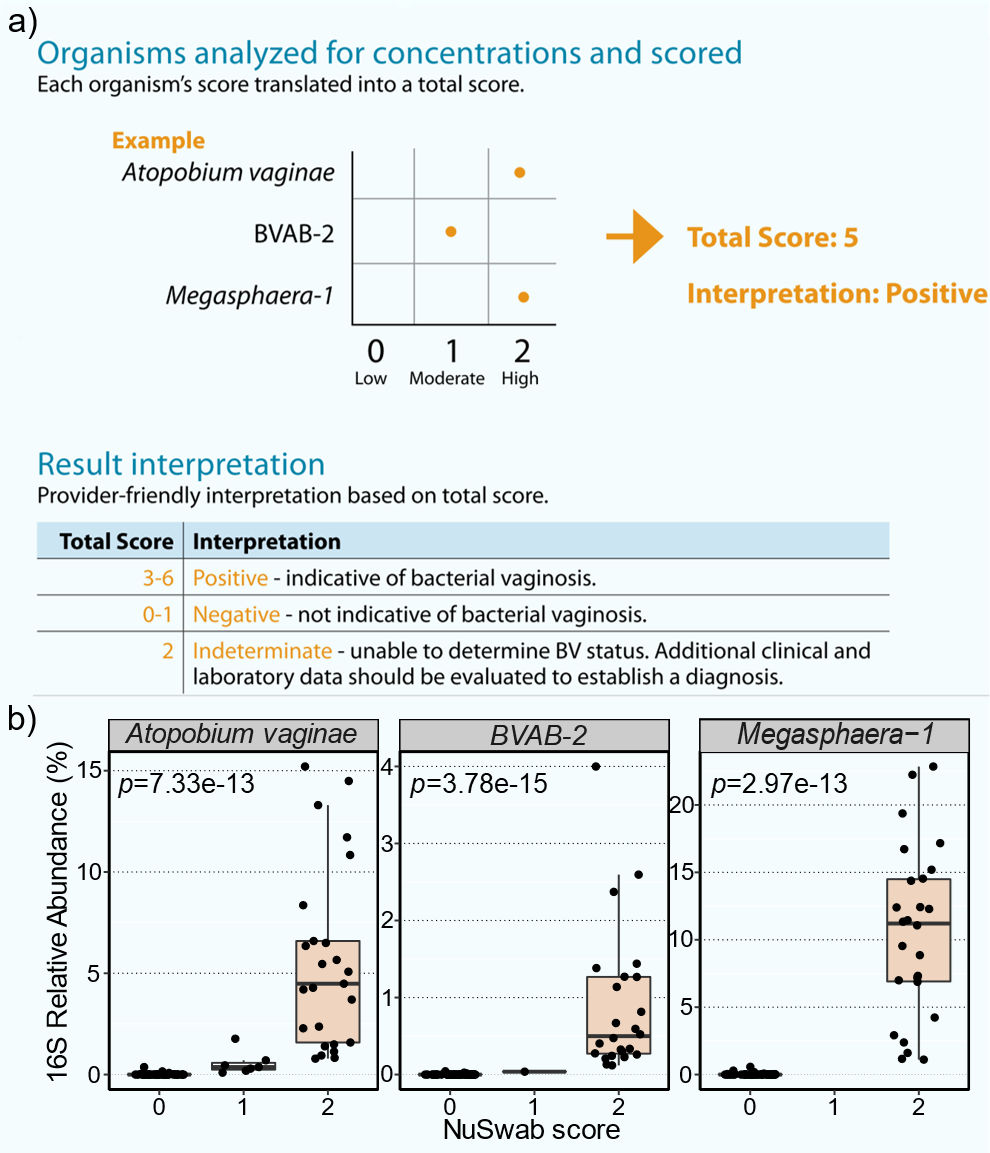
16S V3–V4 rRNA sequencing of remnant clinician-collected vaginal swabs previously analyzed via the Labcorp NuSwab BV PCR test. (**a**) Example process of determining the bacterial vaginosis (BV) status of a vaginal swab using the NuSwab three-microbe panel composed of *Atopobium vaginae*, BVAB-2, and *Megasphaera-1*. Each microbe is quantified and given a score of 0 (low), 1 (moderate), or 2 (high). The total score is then interpreted as BV-POS (3–6), BV-IND (2), or BV-NEG (0–1). (**b**) 16S relative abundances of NuSwab panel microbes stratified by their scores. Statistical comparisons of 16S relative abundances were made between samples with NuSwab scores of 2 and those with scores of 0 or 1 using Wilcoxon rank-sum tests with FDR control.

Seventy-five remnant clinician-collected vaginal swabs were previously analyzed via the Labcorp NuSwab test (10), a PCR-based diagnostic method that detects bacterial vaginosis (BV) by scoring three key BV-associated microbes: *Atopobium vaginae*, *BVAB-2*, and *Megasphaera-1* (Fig. 1a). PCR analysis of these vaginal swabs yielded BV-positive (BV-POS, 27 of 75, 36%), BV-indeterminate (BV-IND, 3 of 75, 4%), and BV-negative (BV-NEG, 45 of 75, 60%) diagnostic results. To further characterize these vaginal swabs, we profiled their microbiomes via 16S V3–V4 rRNA gene sequencing analysis. Sequencing yielded 492 unique 16S V3–V4 ASVs that mapped to 83 unique genera, resulting in a mean of 93.6K genus mapped reads per sample. All three NuSwab-tested microbes were detected among these ASVs, with significant enrichment of 16S relative abundances (RAs) among samples with scores of 2 (Fig. 1b, *P* < 0.001 for each NuSwab-tested microbe, Wilcoxon rank-sum tests). This strong corroboration of BV diagnostic results by 16S sequencing analysis highlights the robustness of the NuSwab test.

**Fig 2:**
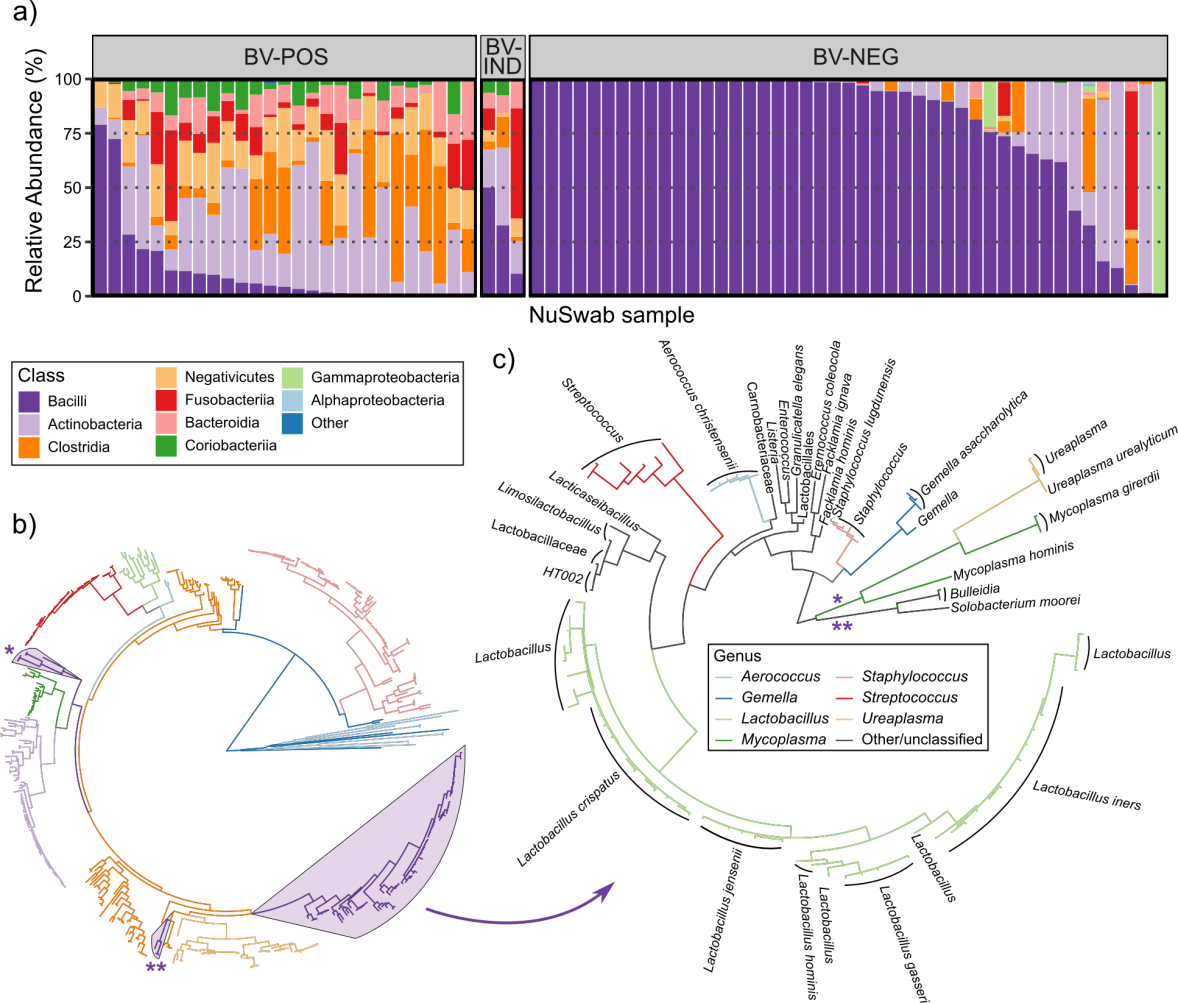
Taxonomic and phylogenetic analysis of 16S V3–V4 amplicon sequence variants (ASVs). (**a**) Stacked bar plot of class relative abundances colored consistently with (**b**) and with samples stratified by BV status. (**b**) ML phylogeny of all ASVs, with branches colored by class. The *Bacilli* class was highlighted and a phylogeny constructed. (**c**) ML phylogeny of ASVs within the *Bacilli* class shown in (**b**) with branches colored by genus and tips labeled with the lowest taxonomic classifications. Neighboring tips with the same label were aggregated into a single label.

We next sought to identify additional characteristics of these vaginal swabs by investigating their ASV-resolved microbiomes. Juxtaposition of samples by BV status using class-level taxonomy revealed a strong propensity for bacilli detection among BV-NEG samples (80.6% mean RA) compared to BV-IND (31%) and BV-POS (11.5%) samples (Fig. 2a). Of the other 10 bacterial classes identified, six had an average RA of 5% or greater in BV-POS and BV-IND samples, while only one of those classes (Actinobacteria) was detected at similar levels in BV-NEG samples (Fig. 2a). This was expected as healthy vaginal microbiomes are known to be heavily populated with *Lactobacilli* (within Bacilli class), while BV microbiomes tend to be more diverse (24). Phylogenetic analysis of ASVs confirmed the accuracy of these taxonomic classifications, with ASVs closely clustering with those assigned to the same class (Fig. 2b). Bacilli ASVs, consistent with their strong prevalence, constituted the plurality of ASVs in the phylogeny (97 of 492, ~ 20%) and formed one large cluster of those predominantly in the *Lactobacillus* genus. Two other smaller bacilli ASV clusters formed mostly comprising *Ureaplasma*, *Mycoplasma*, and *Bulleidia* (Fig. 2b and c). A diverse population of 51 *Lactobacillus* ASVs was detected, most of which were taxonomically classified with species-level resolution (Fig. 2c, 38 of 51, 75%). In total, 13 *L*. *iners*, 11 *L*. *crispatus*, 7 *L*. *jensenii*, 5 *L*. *gasseri*, and 2 *L*. *hominis* ASVs were found, each forming close subgroups in the phylogeny (Fig. 2c). These ASV-resolved, deeply sequenced vaginal microbiomes enable broad assessment of healthy and BV microbiome characteristics, expanding on BV diagnostic insights.

### Characterization of community state types

**Fig 3:**
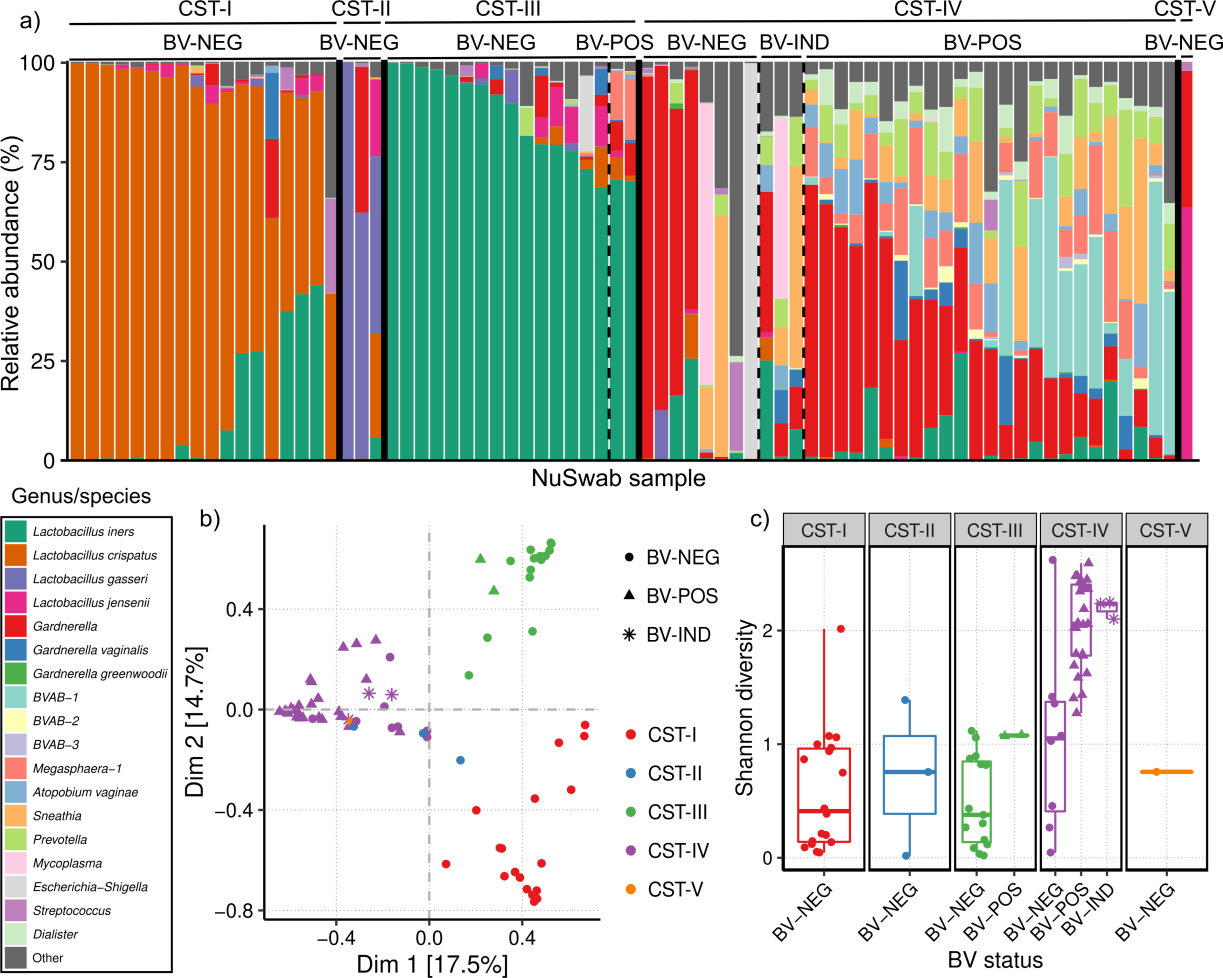
CST analysis of vaginal microbiomes using CST definitions described by Ravel *et al.* (14) (**a**) Stacked bar plot of key genera and species’ relative abundances across CSTs I, II, III, IV, and V. Samples are stratified by CSTs (solid vertical lines) and then by BV status (dashed vertical lines). (**b**) Multidimensional scaling scatter plot of vaginal microbiome Bray–Curtis dissimilarities with data points colored by CST and shaped by BV status. (**c**) Boxplots of vaginal microbiome diversities as measured by Shannon index, stratified first by CST, and then by BV status with data points shaped and colored consistently with those in (**b**).

Next, we further characterized these samples using CST analysis, which differentiates samples by their detection of key *Lactobacillus* species. Specifically, we employed CST analysis per Ravel *et al*. (14), wherein five CSTs are considered based on the most abundant species detected in each sample (CST-I: *L. crispatus*, CST-II: *L. gasseri*, CST-III: *L. iners*, CST-IV: diverse communities, and CST-V: *L. jensenii*). Furthermore, samples without a *Lactobacillus* species exceeding 30% relative abundance were considered diverse (CST-IV). All five CSTs were observed among the 75 NuSwab samples in this study with a strongly significant association between CST and BV status (Table 1, *P* < 0.001, Fisher’s exact test). While most BV-NEG samples were in *Lactobacillus-*dominated CSTs (37 of 45, 82%), BV-POS and BV-IND samples were largely classified in CST-IV (28 of 30, 93%) (Table 1; Fig. 3a). When clustering sample microbiome profiles using multidimensional scaling of their pairwise Bray–Curtis dissimilarities, we observed significant separation of samples by their CST classification (Fig. 3b, *P* < 0.001, PERMANOVA).

**TABLE 1:** Community state type (CST) classifications of NuSwab vaginal samples^*a*^

NuSwab result	CST-I	CST-II	CST-III	CST-IV	CST-V	Total
BV-NEG	18	3	15	8	1	45
BV-IND	0	0	0	3	0	3
BV-POS	0	0	2	25	0	27
Total	18	3	17	36	1	75

^*a*^ Each table entry indicates the number of samples classified in each CST from each of the NuSwab result categories (BV-NEG, BV-IND, and BV-POS). The marginal totals are shown on the right and bottom of the table.

We also observed that BV-POS samples were notably more diverse than BV-NEG samples (Fig. 3c), consistent with their tendency to be within CST-IV (Table 1; Fig. 3a). Interestingly, within CST-IV, we still observed significantly higher microbiome diversity among BV-POS and BV-IND samples compared to BV-NEG samples (Fig. 3c, *P* < 0.001, Wilcoxon rank-sum test). This suggests that vaginal microbiome classification in CST-IV may represent other forms of dysbiosis not necessarily caused by BV. Together, these findings indicate that among these NuSwab samples, BV is associated with diverse, heterogeneous microbiomes that generally lack the essential vaginal *Lactobacilli*.

Some studies argue that the V3–V4 hypervariable region might insufficiently resolve key vaginal *Lactobacillus* species (25, 26) (e.g., *L. iners*); therefore, we confirmed the accuracy of our CST classifications using shotgun metagenomic sequencing (MGx), which has been used to investigate species-refined vaginal microbiome diversity at the population level (27). A separate group of 54 remnant NuSwab vaginal samples were sequenced via both 16S V3–V4 rRNA gene sequencing and MGx, and CST results were generated using the same ruleset as above for both sequencing methods (Table 2). A total of 53 of 54 (98%) samples had concordant CST calls across sequencing methods spanning all five CSTs. Closer inspection of the discrepant CST classification revealed only a difference in *L. crispatus* (16S: 37.1%, MGX: 61.4%) and *L. iners* (16S: 52.8%, MGX: 38.2%) relative abundances. Since *L. crispatus* and *L. iners* are phylogenetically distinct (Fig. 2c), this relative abundance difference was likely due to biases from the sequencing methods rather than 16S misclassification. These results show that our 16S V3–V4 vaginal *Lactobacillus* speciation is robust and capable of yielding accurate CST classifications for vaginal swabs.

**TABLE 2:** Comparison of CST classifications between 16S rRNA gene V3–V4 sequencing and shotgun metagenomic sequencing (MGx)^*a*^

MGx	16S rRNA gene V3–V4				
	CST-I	CST-II	CST-III	CST-IV	CST-V
CST-I	16	0	1	0	0
CST-II	0	1	0	0	0
CST-III	0	0	17	0	0
CST-IV	0	0	0	14	0
CST-V	0	0	0	0	5

^*a*^ Rows and columns indicate CST calls determined from MGx and 16S rRNA gene V3–V4 sequencing results, respectively.

### BV-POS samples are enriched with distinct bacterial networks

**Fig 4:**
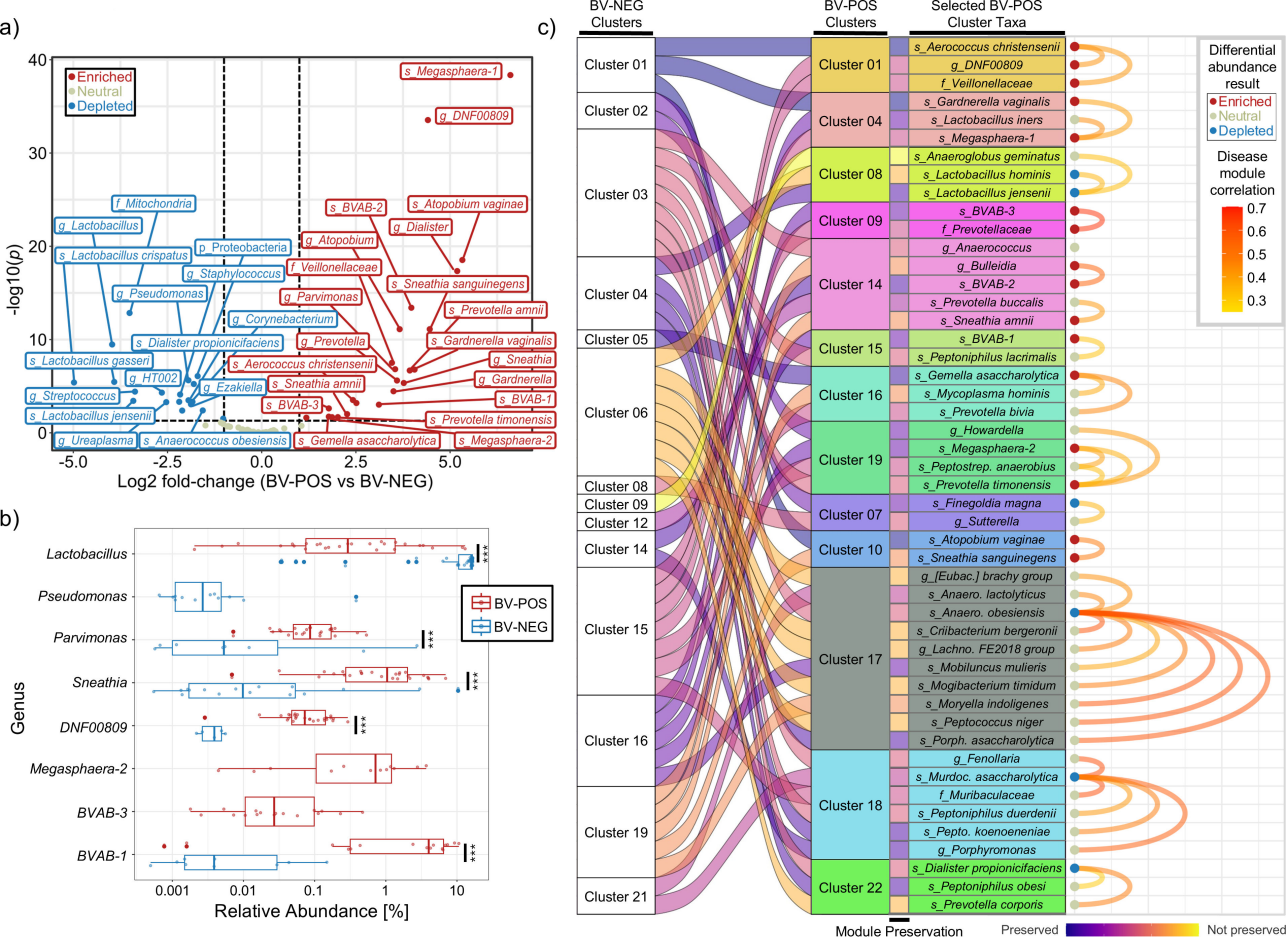
Differential abundance (DA) analysis and modularized co-occurrence network analysis of BV-POS and BV-NEG samples. (**a**) Volcano plot of the differential abundance results, depicting the log2 fold-change (L2FC) (x-axis, BV-POS vs BV-NEG), and the -log10(*P*) (y-axis) for each taxa assessed. FDR control for multiple testing was used to calculate *P* values. Data point colors represent statistical significance of taxa, with red representing enriched taxa (*P* < 0.05, L2FC >= 1), blue representing depleted taxa (*P* < 0.05, L2FC <= −1), and yellow representing all other taxa without significant changes. (**b**) Boxplots comparing the relative abundances of select taxa between BV-POS and BV-NEG samples, with statistical significance indicated to the right (*** *P* < 0.001). Unique identification of taxa is represented by only a single boxplot shown (e.g., *Parvimonas* exclusively detected in BV-NEG samples). (**c**) Clustering results from C3NA (32), with the first column representing clustering among the BV-NEG samples (*n* = 25 clusters), followed by the modular preservation ribbon linked to the colored BV-POS clusters (*n* = 19). The arc on the right represents the Spearman correlations above 0.2 among each BV-POS cluster, with each node colored by the DA result (enriched, neutral, and depleted) and the edge colored by the correlation magnitude. All testing was performed on the species level with any nonspeciated taxa labeled at the highest resolved taxonomic levels, i.e., "g_*DNF000809*" represents the ASVs that resolved to the *DNF000809* genus without species assignment.

Since vaginal microbiomes exhibited clear differences in CST classifications and microbial diversity based on BV status (Fig. 3; Table 1), we systematically analyzed microbial differential abundances between BV-POS and BV-NEG samples. Not surprisingly, we found all three key NuSwab BV-associated microbes significantly enriched and multiple *Lactobacilli* species significantly depleted among BV-POS samples (Fig. 4a). We also detected multiple other known BV-associated microbes enriched in BV-POS samples, including *BVAB-1* (28), *BVAB-3* (28), *Megasphaera-2* (29), *Sneathia* (30), *DNF00809* (29), and *Parvimonas (*31) (Fig. 4a and b).

We also evaluated the modularity of these microbes using a correlation-based analysis that assessed microbe–microbe correlations separately for BV-POS and BV-NEG samples (*Methods*). This approach enabled us to simultaneously evaluate correlation network conservation and whether differentially abundant microbes coordinate with each other based on the BV status. Interestingly, each NuSwab BV-associated microbe was found in a separate correlation-based cluster, suggesting that they broadly capture BV microbial signatures (Fig. 4c). *Atopobium vaginae* formed a small cluster with only *Sneathia sanguinegens* (Cluster 10), while *BVAB-2* clustered with *Sneathia amnii* and *Bulleidia* (Cluster 14) and *Megasphaera-1* clustered with *Gardnerella vaginalis* (Cluster 04) (Fig. 4c). We also identified additional clusters of BV-associated microbes including *DNF00809* (Cluster 01), *BVAB-3* (Cluster 09), BVAB-1 (Cluster 15), and *Megasphaera-2* (Cluster 19) (Fig. 4c). These results indicate that multiple coordinated microbial networks may drive BV pathogenesis and further support the use of multiple diagnostic targets for BV detection.

### Prediction of a BV-associated metabolic signature

In addition to the microbiome, the metabolome of the vaginal microenvironment has known associations with BV pathogenesis and often exhibits immunomodulatory functions (33). To better understand the functional capacity of the BV microbial signatures we identified, we predicted the metabolic pathway abundances from the ASVs using PICRUSt2 (34) and MetaCyc (35) (*Methods*). Since only few samples were classified as CST-II and CST-V (Table 1), we constrained this analysis to CST-I, CST-III, and CST-IV, and we investigated whether metabolic pathways were significantly enriched or depleted (*P* < 0.05) based on BV positivity within and between CSTs. Since all but two BV-POS samples were in CST-IV, only CST-IV BV-POS samples were considered. Comparisons of CST-I and CST-III BV-NEG samples versus the BV-POS samples separately revealed 270 and 232 differentially abundant (DA) metabolic pathways, respectively (Table 3; Supplementary Data). When comparing the CST-IV BV-NEG and CST-IV BV-POS samples, 36 DA metabolic pathways were detected, all of which were detected in the other two comparisons (Table 3; Supplementary Data). Furthermore, a separate comparison of BV-NEG samples between CST-I and CST-III revealed only 39 DA metabolic pathways, which only represented a small fraction of the pathways in the other comparisons (Table 3; Supplementary Data). This indicated that the BV-associated metabolic pathways were largely not due to a deficiency of *Lactobacilli* and were more likely driven by the microbes enriched in BV-POS samples (Fig. 4).

**TABLE 3:** Differential abundance analysis of predicted metabolic pathways between BV-NEG and BV-POS samples^*a*^

BV-NEG	Total	CST-I BV-NEG vs CST-III BV-NEG (39 total)	BV-NEG vs BV-POS		
			Total	Shared	Unique
CST-I	270	36	234	194	40
CST-III	232	30	202	194	8
CST-IV	36	3	33	33	0

^*a*^ Each row shows the number of significantly differentially abundant (DA, *P* < 0.05) metabolic pathways between BV-NEG and BV-POS samples within and between CSTs. In all comparisons, the BV-POS samples were from CST-IV; hence, only the BV-NEG CST is shown in the table. The total number of DA pathways is shown on the left followed by the number of pathways that were also DA between BV-NEG samples compared across CST-I and CST-III. On the right, the remaining pathways are shown with the total followed by the number shared with other comparisons and the number unique to the comparison.

**Fig 5:**
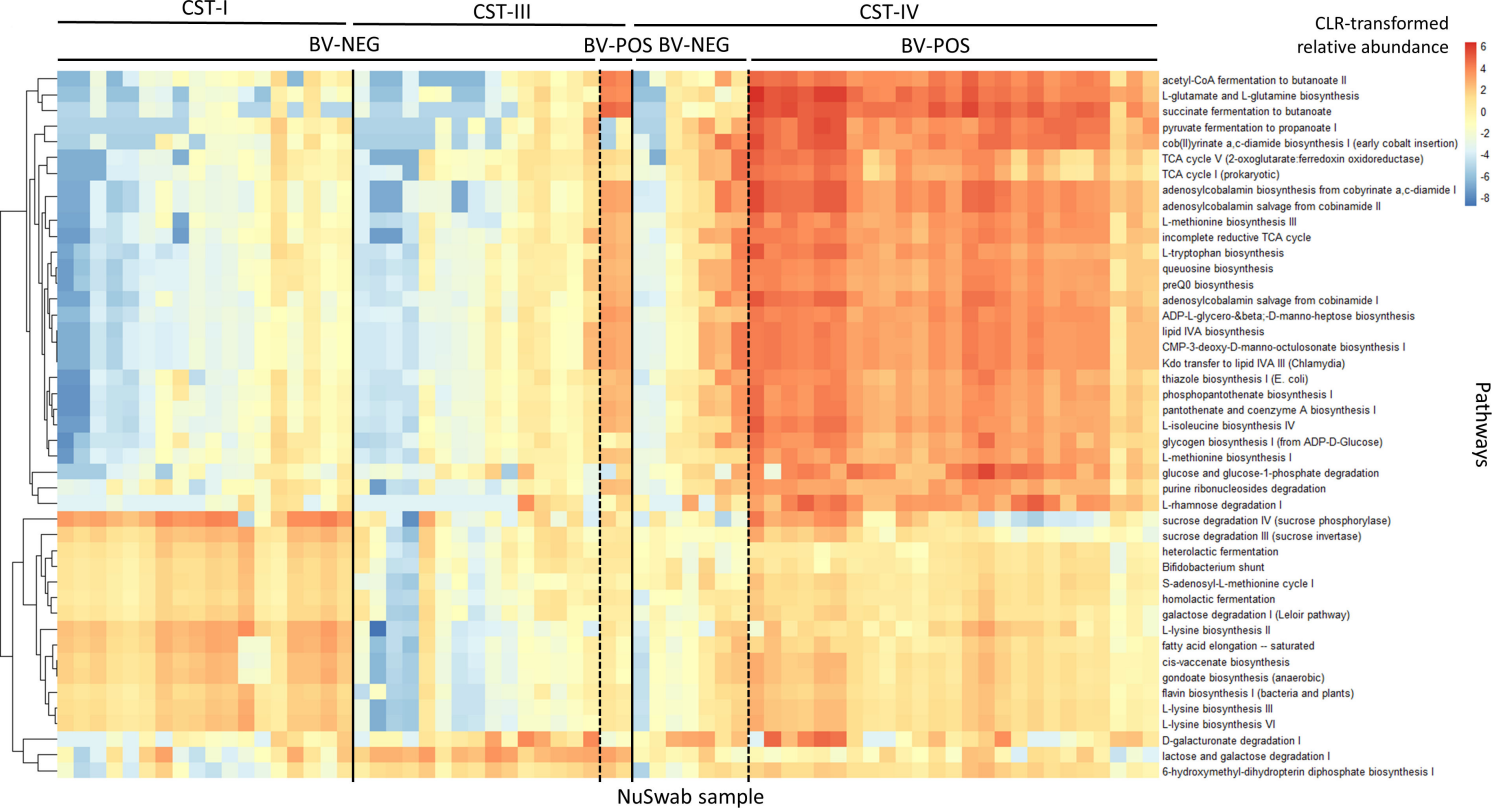
Heatmap of differentially abundant (DA) predicted metabolic pathways between BV-POS and BV-NEG samples found in CST-I, CST-III, and CST-IV. Rows represent MetaCyc (35) metabolic pathways and are ordered via hierarchical clustering, while columns (samples) are stratified first by CST (solid vertical lines) and then by BV status (dashed vertical lines). The heatmap combines the results of four pairwise comparisons, namely (i) CST-I BV-NEG vs CST-III BV-NEG; (ii) CST-I BV-NEG vs CST-IV BV-POS; (iii) CST-III BV-NEG vs CST-IV BV-POS; and (iv) CST-IV BV-POS vs CST-IV BV-NEG. The top 20 differentially abundant pathways of each comparison with the greatest variance were selected and merged into the list of significant pathways across all pairs of sample groups (combined set of 45 pathways shown).

To visually examine the conservation of the four sample comparisons made above (Table 3, including the BV-NEG CST-I versus CST-III comparison), we selected the most variable DA pathways from each comparison and merged them into a single heatmap (Fig. 5). Consistent with our DA results (Table 3; Supplementary Data), BV-POS samples showed highly similar patterns of enriched and depleted pathways regardless of the CST (Fig. 5). Interestingly, the three BV-NEG samples within CST-IV (the three left-most samples within BV-NEG CST-IV in Fig. 5) exhibit a similar pattern of pathways with BV-POS samples. This similarity suggests a potential transition from a healthy state to BV, offering a plausible explanation for some patients experiencing BV symptoms while testing negative. Among the pathways significantly enriched in BV-POS samples in all comparisons with BV-NEG samples, we observed four noteworthy pathways: acetyl-CoA fermentation to butanoate, L-glutamate and L-glutamine biosynthesis, succinate fermentation to butanoate, and pyruvate fermentation to propanoate. These enrichments were indicative of higher concentrations of four known BV-related metabolites, namely, acetate, succinate, butanoate, and propanoate (36, 37). Overall, these findings indicate that BV induces large metabolic disruptions in the vaginal microenvironment.

## DISCUSSION

In this study, we comprehensively profiled the microbial and metabolic signatures of remnant clinician-collected, PCR-tested healthy and BV sample microbiomes. We demonstrated that 16S V3–V4 rRNA gene sequencing can (i) reproduce Labcorp NuSwab BV PCR testing results; (ii) accurately classify vaginal microbiome CSTs via *Lactobacillus* speciation; and (iii) identify additional microbial and metabolic correlates of BV positivity.

By comprehensively profiling the vaginal microbiome with ASV-level resolution, we elucidated multiple features of BV positivity and confirmed many previous findings. Not surprisingly, we found that *Lactobacillus* species were less abundant among BV-POS samples relative to BV-NEG samples. Moreover, BV-POS samples were significantly more diverse and enriched with multiple BV-associated bacteria, such as *Gardnerella* (including *G. vaginalis*), *Prevotella*, *Sneathia*, *Dialister*, *BVAB-1*, *BVAB-3, Veillonellaceae*, *DNF00809*, *Aerococcus christensenii, Parvimonas,* and *Bulleidia* (28, 29, 38–41). The three NuSwab BV-associated microbes (*Atopobium vaginae*, *BVAB-2*, and *Megasphaera-1*) were also among the enriched bacteria in BV-POS samples. Interestingly, we observed weak enrichment of *Gemella asaccharolytic*a in BV-POS samples, whereas *Gemella* species have previously been less frequently detected in BV-POS samples (28). We also uniquely evaluated the correlation networks of BV-associated bacteria using C3NA (32), highlighting key modular differences between the BV-POS and BV-NEG samples in this study. We uncovered multiple clusters of BV-associated microbes, and intriguingly the three NuSwab BV-associated microbes were identified in three separate clusters. This indicates that the NuSwab PCR test robustly quantifies biomarkers from different BV microbial networks, consistent with the strong diagnostic utility previously reported for the NuSwab test in a multicenter study (11).

In this study, we also interrogated the metabolic signatures that distinguish vaginal microbiomes based on their CST and/or BV diagnostic status. We observed four noteworthy pathways: acetyl-CoA fermentation to butanoate, L-glutamate and L-glutamine biosynthesis, succinate fermentation to butanoate, and pyruvate fermentation to propanoate. The enrichment of these pathways among BV-POS samples indicated higher concentrations of four known BV-related metabolites, namely, acetate, succinate, butanoate, and propanoate (36, 37). Acetate and butanoate are known to play a role in immune responses (42). We also observed three pathways significantly depleted within CST-I, namely, D-galacturonate degradation, lactose and galactose degradation, and 6-hydroxymethyl-dihydropterin diphosphate biosynthesis, and some pathways significantly depleted in CST-III, such as sucrose degradation and L-lysine biosynthesis. The observed depletion of L-lysine biosynthesis in CST-III corresponds with findings from prior studies, which have indicated that *L. iners*, the dominant species in CST-III, has a considerably smaller core genome for the biosynthesis of lysine compared to *L. crispatus*, the dominant species in CST-I (43). While these findings support potential BV metabolic biomarkers, more rigorous multiomic studies that also interrogate host metabolites will be needed to establish a mechanism. For example, a recent study analyzed the vaginal metabolomes of African women diagnosed with BV and identified the rapamycin (mTOR) signaling pathway as a signature of BV-induced epithelial dysfunction (44). Other approaches, such as those that simultaneously quantify host and microbial RNA (45), could also improve our understanding of host–microbe interactions during BV pathogenesis.

In recent years, the taxonomy of *Gardnerella* has undergone nomenclature revisions, resulting in the naming of five new species: *G. swidsinskii*, *G. leopoldii*, *G. pickettii*, *G. piotii*, and *G. greenwoodii* (46, 47). In this study, we applied extra rigor in our taxonomic assignment of *Gardnerella* ASVs, successfully resolving *G. vaginalis* and *G. greenwoodii*. Unfortunately, many ASVs were unable to be resolved at the species level, highlighting a key limitation of the 16S V3–V4 region for assessment of diverse *Gardnerella* populations. However, we still identified *G. vaginalis* and a broad group of unresolved *Gardnerella* as enriched among BV-POS samples. These findings were supported by recent developments of PCR panels for *Gardnerella* subtyping, which showed enrichment of multiple *Gardnerella* species among individuals with BV (48). While *G. vaginalis* is targeted by some commercially available PCR tests (49), its diagnostic utility remains unclear, with some studies showing a lack of BV association (50). Species-level resolution of *Gardnerella* populations is needed to resolve these disagreements in the literature. For example, a recent study using shotgun metagenomic sequencing of vaginal microbiomes showed differences in *Gardnerella* species richness across multiple cohorts of pregnant women (51). Metagenome-assembled genomes (MAGs) are an effective strategy for improving taxonomies, with a recent study utilizing MAGs to interrogate the early-life gut microbiome (52). High-quality MAGs from long-read sequencing technologies have proven especially effective in resolving closely related strains (53), but to our knowledge have not yet been applied to vaginal microbiomes. Furthermore, dedicated vaginal microbiome database developments, such as the Isala citizen-science project (16) and the Vaginal Microbial Genome Collection (VMGC) (54), are critical for improved vaginal microbiome taxonomic resolution. Multiplex PCR and sequencing-based diagnostics for BV and other forms of vaginal dysbiosis will greatly benefit from the curation of such strain-resolved genomic resources.

Multiple factors must be considered as we move to implement clinical sequencing-based diagnostics, namely, limitations in the data resolution, sequencing costs, and the interpretation of results. If employing traditional short-read 16S sequencing, one must consider the limitations in data resolution offered by the chosen hypervariable regions (V3–V4 in our case) as hypervariable regions are known to yield differences in microbiome profiling resolution (55). Fortunately, we show that the V3–V4 hypervariable regions accurately speciate key strains of *Lactobacillus*, but other genera, such as *Gardnerella*, had fewer species resolved. Thus, limitations in 16S rRNA amplicon taxonomic resolution could be a barrier for clinical use. However, full-length 16S rRNA gene sequencing has been shown to improve taxonomic resolution and resolve single-nucleotide differences in the entire 16S rRNA gene (56). In practice, full-length 16S rRNA gene sequencing has already been shown to significantly improve species-level accuracy using more than one sequencing platform, highlighting its potential clinical utility (57–60). Moreover, a recent study showcased a bioinformatics workflow tailored for long-read 16S rRNA gene sequencing of vaginal microbiomes suitable in a clinical setting (61). Shotgun metagenomic and metatranscriptomic sequencing have also provided highly resolved insights into the vaginal microenvironment (62), including fungi, parasites, and DNA viruses, suggesting potential for clinical diagnostics as well. However, the relatively lower cost of amplicon sequencing and the lack of reliance on complete assembled genomes might lower the barrier to entry for long-read 16S rRNA gene sequencing as a clinical diagnostic tool. Recent advances in cDNA concatenation techniques now enable higher-throughput (and lower cost) long-read 16S rRNA gene sequencing (63). Furthermore, the cost-effectiveness of 16S rRNA gene sequencing can be optimized in the clinic via intelligent design of diagnostic reflex testing from PCR tests (e.g., Labcorp NuSwab test) (64). Large data sets of 16S rRNA ASVs from various microbes are reproducible and thus an attractive choice for developing possible specific microbiome-based biomarkers for human diseases where the role of the microbiome has already been well established, such as cancer (65–67) and diabetes mellitus (68–70). In this study, we explore the feasibility of 16S rRNA gene sequencing and largely adhere to recent guidelines for clinical microbiome analysis (71), showcasing the potential for clinical validation with a larger population cohort in the future.

Multiple commercially available tests exist for diagnosing BV in symptomatic women with no recommendations for those who are asymptomatic (49). Amsel’s criteria and Nugent score are mainstays of BV diagnosis, but their application may vary in clinical settings due to their subjective nature and technician dependency (72). Multiplex PCR tests offer stronger sensitivity and specificity at greater expense than Nugent score and Amsel’s criteria, but differ in the microbes that they target (49). This variability in BV diagnostics poses a challenge for uncovering novel BV biomarkers, which can be overcome using sequencing-based technologies. In this study, we confirmed the robustness of 16S rRNA gene sequencing of BV-tested remnant vaginal swabs (Labcorp NuSwab test) collected from a U.S.-based population. However, future investigations of diverse populations outside the United States are needed to evaluate and refine the BV biomarkers we described. Also, further work is needed to identify BV microbial and metabolic biomarkers from unconventional clinical manifestations of BV, such as from women who suffer from recurrent BV or from those who present with symptoms but are PCR-negative. Large prospective cohort studies of individuals afflicted with recurrent BV symptoms utilizing clinically available PCR testing and 16S rRNA gene-based microbiome profiling (and potentially metagenomic sequencing as well) will be invaluable in helping to close these gaps in the care and treatment of BV.

In this study, we profiled the vaginal microbiomes of remnant vaginal swabs clinically tested using the Labcorp NuSwab BV PCR test. Our findings support the accuracy of NuSwab in identifying BV and provide valuable insights for advancing the diagnostic and treatment options available to patients. These results highlight the potential for 16S rRNA gene profiling in the diagnosis of BV.

## MATERIALS AND METHODS

### Sample collection, 16S V3–V4 rRNA gene sequencing, and shotgun metagenomic sequencing

Seventy-five de-identified clinician-collected remnant NuSwab samples were procured internally at Labcorp. These samples originated from patients throughout the continental United States, and patient demographic data were not available for use in this study. All samples were originally collected through the NuSwab test using either an Aptima Multitest Swab Specimen Collection Kit or an Aptima Unisex Swab Specimen Collection Kit and were stored at room temperature. These samples underwent DNA extraction using the ZymoBIOMICS Magbead DNA isolation kit. The 16S rRNA gene V3–V4 hypervariable region was amplified using the 341 f (5′-CCTACGGGNGGCWGCAG-3′) and 805 r (5′-GACTACHVGGGTATCTAATCC-3′) primers (73). Library preparation was performed using KAPA HyperPrep and KAPA Library amplification kits, and the resulting libraries were sequenced on an Illumina MiSeq platform using 300-bp paired-end reads. A separate set of 54 remnant vaginal swabs were processed through the aforementioned sequencing workflow and through a shotgun metagenomic assay comprising total microbial DNA extraction via the ZymoBIOMICS Magbead DNA isolation kit, library preparation using the KAPA HyperPlus Library Preparation Kit, and sequencing on the Illumina NextSeq2000 platform using 150-bp paired-end reads.

### DADA2 processing and taxonomic analysis

16S rRNA gene V3–V4 sequencing reads were processed using R v4.1.1 (74) by first reorienting reads to the same strand and then trimming using the DADA2 v1.22.0 (75) filterAndTrim function with default parameters and trimRight = c (52, 68) (optimally chosen using in-house R function). Quality-filtered trimmed reads were then processed through the standard DADA2 (75) process, denoising, and merging reads to generate amplicon sequence variants (ASVs). ASVs were then filtered by removing singletons and those with lengths shorter than 350 bp. The resulting ASVs were taxonomically classified using the SILVA v138.1 database (76) (training data formatted for DADA2 obtained from Zenodo, DOI: 10.5281/zenodo.4587955) and DADA2-implemented RDP classifier (77) with a bootstrap confidence threshold of 80. Specifically, the DADA2 assignTaxonomy function was used with minBoot = 80 and the silva_nr99_v138.1_train_set.fa.gz file, followed by the addSpecies function using default parameters and the silva_species_assignment_v138.1.fa.gz file. ASVs without at least a phylum rank classification were removed from analysis.

Additional ASV speciation was performed by aligning ASVs with a genus rank but no species rank classification directly to the SILVA v138.1 database (76) using the VSEARCH v2.17.1 (78) usearch_global function with parameters “--id 0.99—strand both—maxgaps 0—minwordmatches 0—maxaccepts 0.” Aligned ASVs were assigned to a species if all optimal alignments matched to a single species. *Lactobacillus*-focused speciation was then performed by 99% clustering of all ASVs classified in the *Lactobacillus* genus using CD-HIT v4.8.1 (79) with parameter -c 0.99. ASVs found in clusters with ASVs previously taxonomically assigned to only a single *Lactobacillus* species (i.e., via RDP classification or direct alignment) were assigned to the same *Lactobacillus* species. Since the NuSwab BV assay targets microbes not in the SILVA nomenclature (*BVAB-2*) and with phylotype resolution (*Megasphaera-1*), ASVs with genus rank classification were further taxonomically analyzed by direct alignment using VSEARCH v2.17.1, as described above to a custom database of 16S sequences obtained from the Bacterial and Viral Bioinformatics Resource Center (BV-BRC) (80) and additional *BVAB-2* and *Megasphaera* phylotype reference sequences (81, 82). ASVs with uniquely optimal alignments to *BVAB-1*, *BVAB-2*, *BVAB-3*, *Megasphaera-1*, or *Megasphaera-2* were assigned accordingly at the species rank.

Lastly, to accurately assign *Gardnerella* species not in the SILVA v138.1 database (76), all 132 Refseq-annotated *Gardnerella* genomes were obtained from the NCBI Genome Dataset (83) (https://www.ncbi.nlm.nih.gov/datasets/genome/, accessed 11 December 2024). Full-length 16S sequences of recently designated *Gardnerella* species were also included (46). 16S V3–V4 sequences were extracted *in silico* by aligning the 341 f and 805 r primers (see previous section) to these reference sequences using VSEARCH v2.17.1 as described above. Extracted sequences were confirmed as *Gardnerella* using RDP classification described above. Sequences shared across multiple and/or unnamed *Gardnerella* species genomes were labeled as *Gardnerella*, while those unique to a species retained the species label. All ASVs assigned to the *Gardnerella* genus were then aligned to the extracted sequences using VSEARCH v2.17.1, as described above. ASVs with optimal alignments to a single *Gardnerella* species were assigned accordingly at the species rank, while those optimally aligned to multiple species and/or sequences labeled as *Gardnerella* were left unassigned at the species rank. A final taxonomically aggregated count matrix was then generated by aggregating ASVs at the lowest assigned taxonomic rank.

### Phylogenetic, diversity, and community state type analysis

Phylogenetic analysis was performed by generating a multiple sequence alignment of all ASVs using Clustal Omega v1.2.4 (84) and processing it in R v4.1.1 using the phangorn v2.8.1 package (85). A neighboring joining tree was constructed using the dist.ml and NJ functions. Then, a Jukes–Cantor optimized maximum likelihood tree was generated using the pml and optim.pml functions with optNni = T. The ggtree v3.2.0 R package (86) was used to visualize the resulting phylogenies.

Sample alpha diversity and community state types (CST) were determined using the final taxonomically aggregated count matrix (see Section “DADA2 processing and taxonomic analysis”). Alpha diversity was calculated using the diversity function in the vegan v2.6–2 R package (87). CSTs were determined using the most abundant taxon detected, following the criteria used by Ravel *et al. (*14) (CST-I: *L. crispatus*, CST-II: *L. gasseri*, CST-III: *L. iners*, CST-IV: diverse communities, and CST-V: *L. jensenii*). Furthermore, samples without a *Lactobacillus* species exceeding 30% relative abundance were considered diverse (CST-IV).

### Confirmation of 16S rRNA gene-resolved CSTs via shotgun metagenomic sequencing

To validate the 16S-based CST characterization, an additional set of 54 remnant vaginal swabs were internally procured and sequenced via 16S rRNA gene V3–V4 sequencing as described above and via shotgun metagenomics (MGx). The CST classification obtained from the 16S and MGx were compared to validate the reliability of CST types from the 16S pipeline. The MGx reads were processed using Illumina bcl2fastq v2.2 and then filtered using BBMAP v38.98 (88) to ensure high-quality reads with a minimal length of 100 bp. Taxonomic assignments were performed using Kraken v2.1.2 (89), followed by Bracken v2.7 (90) using the pre-built standard plus Refseq protozoa and Fungi (PlusPF) database. CSTs were determined as described above in Section “Phylogenetic, diversity, and Community State Type analysis” using the most abundant species detected from the Bracken read re-assignment output (MGx) and the most abundant taxon from the 16S data processing.

### Differential abundance analysis of microbes

The differential abundance analysis of microbes between BV-POS and BV-NEG samples was performed by using the ANCOM-BC v2.0.3 R package (91). The three BV-indeterminant samples were excluded. Significantly enriched taxa were identified with FDR-adjusted *P* < 0.05 and log2 fold-change (L2FC) >=1. Significantly depleted taxa were identified with FDR-adjusted *P* < 0.05 and L2FC <= −1. The remaining taxa were identified as neutral (FDR-adjusted *P* >= 0.05 or –1 < L2 FC <1).

### Modularized co-occurrence network analysis

BV-POS and BV-NEG samples were processed using C3NA v1.0.0 (32) to perform modularized co-occurrence network analysis. To account for the cross-taxonomy design and the unique microbial compositional structure, all cross-taxonomy correlations were obtained using FastSpar v1.0.0 (92), a C ++ implementation of Sparse Correlation for Compositional data (SparCC) (93). Then, the optimal number of taxa clusters was separately determined for BV-POS and BV-NEG samples using a consensus approach implemented in C3NA v1.0.0 (32).

### Prediction and differential abundance analysis of metabolic pathways

Two CSTs with insufficient samples, CST-II with three samples and CST-IV with one sample, were excluded in the metabolic pathway prediction. One outlier BV-NEG sample in CST-IV (NS43) and all three BV-indeterminate samples were excluded. The remaining 67 samples from three major CSTs, CST-I, CST-III, and CST-IV, were used to perform metabolic pathway prediction. The MetaCyc (35) metabolic pathways of microbes in samples were predicted using their 16S rRNA gene sequences using PICRUSt2 v2.5.0 (34) run with default options. Then, the ggpicrust2 v1.7.3 R package (94) was used to identify differentially abundant pathways between different groups of samples. Specifically, the ggpicrust2 pathway_daa wrapper function was used to perform Wilcoxon rank-sum tests, which utilized the aldex.clr and aldex.ttest functions in the ALDEx2 v1.32.0 R package (95) with default parameters. The differential abundance analysis of metabolic pathways was performed on four pairs of groups of samples, namely, BV-NEG samples within CST-I and BV-POS samples within CST-IV, BV-NEG samples within CST-III vs BV-POS samples within CST-IV, BV-NEG samples within CST-I vs BV-POS samples within CST-IV, BV-NEG samples within CST-I vs BV-NEG samples within CST-III, and BV-NEG samples within CST-IV vs BV-POS samples within CST-IV. Significantly differentially abundant features among different groups of samples were identified using an FDR-adjusted *P* < 0.05. Differentially abundant pathways were visualized using their centered log-ratio-transformed scores and the pheatmap v1.0.12 R package (96). Any pathways described as “superpathways” or “engineered” were excluded from the heatmap.

### Statistical analysis

The relationship between sample BV status and CST classification was examined using Fisher’s exact tests using the fisher.exact function in R v4.1.1 (74). Differences in microbiome alpha diversity based on BV status and CST were examined using Wilcoxon rank-sum tests using the wilcox.test function in R v4.1.1 (74). Statistical significance of sample CST clustering in the first two dimensions of multidimensional scaling (MDS) was performed using permutational multivariate analysis of variance (PERMANOVA). In all analyses where multiple hypotheses were assessed, false discovery rate (FDR) correction was performed using the Benjamini & Hochberg procedure.

## Data Availability

Software used in this study, count matrices, sample metadata and NuSwab test results, ASV taxonomy and sequences, PICRUSt2 predicted pathway abundances, ANCOM-BC microbial differential abundance results, and ALDEx2 pathway differential abundance results are provided in the Supplementary Data. Raw 16S V3–V4 rRNA gene sequencing data are available under BioProject accession number PRJNA1171405 in the NCBI BioProject database (https://www.ncbi.nlm.nih.gov/bioproject/).

## SUPPLEMENTAL MATERIAL

Supplemental data spectrum.02582-24-s0001.xlsx Processed data and differential abundance results.

ASM does not own the copyrights to Supplemental Material that may be linked to, or accessed through, an article. The authors have granted ASM a non-exclusive, world-wide license to publish the Supplemental Material files. Please contact the corresponding author directly for reuse.
